# Comparison between morphometry and radiomics: detecting normal brain aging based on grey matter

**DOI:** 10.3389/fnagi.2024.1366780

**Published:** 2024-04-15

**Authors:** Yuting Yan, Xiaodong He, Yuyun Xu, Jiaxuan Peng, Fanfan Zhao, Yuan Shao

**Affiliations:** Center for Rehabilitation Medicine, Department of Radiology, Zhejiang Provincial People’s Hospital (Affiliated People’s Hospital), Hangzhou Medical College, Hangzhou, Zhejiang, China

**Keywords:** normal aging, grey matter, morphometry, radiomics, magnetic resonance imaging

## Abstract

**Objective:**

Voxel-based morphometry (VBM), surface-based morphometry (SBM), and radiomics are widely used in the field of neuroimage analysis, while it is still unclear that the performance comparison between traditional morphometry and emerging radiomics methods in diagnosing brain aging. In this study, we aimed to develop a VBM-SBM model and a radiomics model for brain aging based on cognitively normal (CN) individuals and compare their performance to explore both methods’ strengths, weaknesses, and relationships.

**Methods:**

967 CN participants were included in this study. Subjects were classified into the middle-aged group (*n* = 302) and the old-aged group (*n* = 665) according to the age of 66. The data of 360 subjects from the Alzheimer’s Disease Neuroimaging Initiative were used for training and internal test of the VBM-SBM and radiomics models, and the data of 607 subjects from the Australian Imaging, Biomarker and Lifestyle, the National Alzheimer’s Coordinating Center, and the Parkinson’s Progression Markers Initiative databases were used for the external tests. Logistics regression participated in the construction of both models. The area under the receiver operating characteristic curve (AUC), sensitivity, specificity, accuracy, positive predictive value, and negative predictive value were used to evaluate the two model performances. The DeLong test was used to compare the differences in AUCs between models. The Spearman correlation analysis was used to observe the correlations between age, VBM-SBM parameters, and radiomics features.

**Results:**

The AUCs of the VBM-SBM model and radiomics model were 0.697 and 0.778 in the training set (*p* = 0.018), 0.640 and 0.789 in the internal test set (*p* = 0.007), 0.736 and 0.737 in the AIBL test set (*p* = 0.972), 0.746 and 0.838 in the NACC test set (*p* < 0.001), and 0.701 and 0.830 in the PPMI test set (*p* = 0.036). Weak correlations were observed between VBM-SBM parameters and radiomics features (*p* < 0.05).

**Conclusion:**

The radiomics model achieved better performance than the VBM-SBM model. Radiomics provides a good option for researchers who prioritize performance and generalization, whereas VBM-SBM is more suitable for those who emphasize interpretability and clinical practice.

## 1 Introduction

The human brain structure changes with age throughout the lifetime (Ziegler et al., 2012). The atrophy of grey matter (GM) is commonly observed in normal brain aging, accompanied by the shrinkage of white matter volumes and enlargement of the cerebrospinal fluid spaces (Lemaitre et al., 2012). Trajectories of brain aging differ from individual, and aged brains are more prone to cognitive decline (Fjell et al., 2014). Thus, in the past few decades, researchers have continuously studied the macroscopic and microscopic manifestations of brain aging to varying degrees.

Voxel-based morphometry (VBM) and surface-based morphometry (SBM) are common approaches to studying brain morphological changes from the macroscopic level. VBM serves to estimate brain region volumes, such as grey matter volume (GMV) (Weise et al., 2019). SBM is applied to estimate a range of surface features, for example, cortical thickness (CTh), sulcal depth (SD), gyrification index (GI), as well as fractal dimension (FD) (Bachmann et al., 2023). These parameters can help distinguish between groups of controls and patients with neurological and psychiatric disorders (Nickel et al., 2019; Zhao et al., 2022; Ziukelis et al., 2022; Zhang et al., 2023). Previous cross-sectional and longitudinal studies have provided insights into brain region differences in morphological parameters of normal brain aging (Leong et al., 2017; Shen et al., 2018; Aljondi et al., 2019; Zheng et al., 2019; Lamballais et al., 2020). Research has also recommended that the combined use of VBM and SBM could better understand the brain neurobiological processes and improve the accuracy of morphological change detection (Goto et al., 2022).

Radiomics, as a rapidly developing field, can extract quantitative features from medical images to build diagnosis or prediction models to analyze microscopic information (Mayerhoefer et al., 2020). Radiomics features consist of region of interest (ROI) characteristics such as shape, first-order, and texture features, which can obtain a variety of unknown information from different modality images (Bang et al., 2021). The established disease-specific models could be potentially applied to solve clinical problems (Huang et al., 2023). In the area of neurodegenerative disorders, multiple radiomics models have been developed to diagnose mild cognitive impairment, Alzheimer’s disease, and Parkinson’s disease, and predict their progression and treatment effect (Bian et al., 2023; Shahidi et al., 2023).

Both VBM-SBM and radiomics are mainstream methods for neuroimaging analysis. The former focuses on comparing the macrostructural differences in brain regions to distinguish changes in diseases and the latter selects the most representative and meaningful features to build classification models. Although both methods are widely applied in neurodegenerative diseases, there is a lack of research comparing their performance in assessing brain aging. Therefore, this study aimed to construct a VBM-SBM model and a radiomics model based on the GM of cognitively normal (CN) individuals and compare the performance of the two models in identifying the degree of normal brain aging. By analyzing their strengths, weaknesses, and associations, our research could provide a reference for the selection and applicable situation of the two methods for future research.

## 2 Materials and methods

### 2.1 Participants and MRI acquisition

A total of 967 CN subjects were included in this study. Among them, 360 participants were collected from the Alzheimer’s Disease Neuroimaging Initiative (ADNI) database^1^ as the internal dataset. For the external test datasets, 263, 239, and 105 CN subjects were collected from the Australian Imaging, Biomarker and Lifestyle (AIBL) database,^2^ and the National Alzheimer’s Coordinating Center (NACC) database,^3^ and the Parkinson’s Progression Markers Initiative (PPMI) database,^4^ respectively. The four databases are multisite, longitudinal, and open-access large databases. ADNI, AIBL and NACC consist of clinical, cognitive and imaging data and aim to develop biomarkers for tracking brain aging and early detecting Alzheimer’s disease. PPMI was launched to provide comprehensive and standardized data and further identify biological markers of Parkinson’s risk, onset, and progression. Each participating site in the four databases had obtained approval from the ethics committee and informed written consent from participants was conducted following to the Declaration of Helsinki. Subjects were classified into the middle-aged and old-aged groups according to the age of 66. The internal dataset was randomly divided into the training set and internal test set at a ratio of 7:3. All participants had T1-weighted imaging (T1WI) acquired by 3T scanners with the sequence of volumetric three-dimensional magnetization-prepared rapid gradient-echo (3D-MPRAGE) or similar schemes. More details about image acquisition protocols were available on the databases’ websites.

### 2.2 Data processing and model construction

#### 2.2.1 VBM and SBM

SPM12^5^ and CAT12^6^ were employed for VBM and SBM analysis (Weise et al., 2019). Both software tools were run in MATLAB R2016a platform. T1WI DICOM data were converted into NIFTI format via dcm2nii software.^7^ The structural imaging data were segmented with CAT 12, and CTh and central surface data were extracted simultaneously. During the segmentation process, affine regularization, correction for bias-field inhomogeneity, and spatial normalization with the Montreal Neurological Institute (MNI) template were used to remove bias. Total intracranial volumes (TIV) were obtained. No scans were excluded due to poor quality. For VBM, the GM data were smoothed with an 8 mm full width at half maximum (FWHM) of the Gaussian kernel. For SBM, additional surface parameters, including SD, GI, and FD were extracted using the CAT12 surface tools section. Then CTh data were smoothed with a 15 mm FWHM of the Gaussian kernel and other surface parameters data with a 20 mm FWHM of the Gaussian kernel.

The smoothed data were used to perform two-sample t-tests for statistical comparisons between the middle-aged and old-aged groups for GMV, CTh, SD, GI, and FD, respectively, in the CAT12 and SPM12 statistical modules. TIV served as a covariate for VBM analysis to correct different brain sizes. Sex, education, and MMSE were included for both VBM and SBM analyses. The threshold of *p* < 0.05 family-wise error (FWE) correction was applied for VBM and SBM analyses. VBM results saved from CAT12 were loaded into the xjView toolkit^8^ and differential brain ROIs with the Anatomical Automatic Labeling (AAL) atlas were generated by the xjView report section. SBM results were loaded into the CAT12 result presentation section and differential brain ROIs with the Desikan-Killiany (DK40) atlas were generated by the atlas labeling section. Then, the values of GMV, CTh, SD, GI, and FD inside ROI were estimated. All procedures were carried out according to standard protocol,^9^ applying default settings unless indicated otherwise.

Differential brain ROIs with cluster size >50 for GMV, and cluster size >50 with overlap of brain region >40% for CTh, SD, GI, and FD were selected as potential variables. Univariate and multivariable logistic regression were used to build the VBM-SBM model in the training set. Model testing was performed with the internal test, AIBL, NACC, and PPMI test sets.

#### 2.2.2 Radiomics

SPM12 software (see text footnote 5) was used to automatically segment GM from T1WI data. An experienced neuroradiologist who was blinded to the clinical data, examined segmentations and manually modified unsatisfactory cases using ITK-SNAP software.^10^ PyRadiomics (version 3.0) was applied to extract radiomics features of the GM segmentation, which conformed to the Image Biomarker Standardization Initiative guideline (Zwanenburg et al., 2020). Radiomics features were obtained from the GM segmentation of each subject, including shape, first-order, and texture.

The batch effect of different datasets was reduced by using the ComBat method to normalize and gather the data distributions. The development of the radiomics model was on FeAture Explorer (FAE V 0.3.6) platform, a PyRadiomics-based software (Song et al., 2020). The process included feature redundancy with the Pearson Correlation Coefficient value >0.99, feature selection with Analysis of Variance, classifier with Logistic Regression, and 5-fold cross-validation on the training data set to determine the hyper-parameter. The range of the feature number was set from 1 to 10. To find the simplest model and avoid overfitting, the model was determined according to one-standard error criterion which selected the least number of features and an area under the receiver operating characteristic curve (AUC) value within one standard deviation from the highest AUC in the cross-validation set (Gareth et al., 2013). The internal test, AIBL, NACC, and PPMI test sets were used to evaluate the radiomics model.

### 2.3 Statistical analysis

Data analysis was performed with SPSS (version 25.0) and Microsoft Excel 2020. Two-tailed *p* < 0.05 was considered statistically significant. For continuous variables, the Student’s *t*-test and Mann–Whitney test were used to compare normally and nonnormally distributed data, respectively. The Chi-squared test was implemented for categorical variables. Statistical analysis of VBM and SBM data processing was performed with SPM 12 and CAT12 statistical modules, which have been described in detail in the 2.2.1 VBM and SBM part. In the univariate logistic regression analysis, the variables with *p* < 0.05 were selected and input to the stepwise forward multivariable logistic regression to obtain the final brain regions for building the VBM-SBM model in the training set. The AUC, sensitivity, specificity, accuracy, positive predictive value, and negative predictive value were used to evaluate the two model performances. The DeLong test was used to compare the differences in AUCs. The Spearman correlation analysis was used to observe the correlations between age, VBM-SBM parameters, and radiomics features.

## 3 Results

### 3.1 Clinical characteristics

The study included 967 CN participants, of whom 302 participants were classified into the middle-aged group and 665 participants were classified into the old-aged group. Supplementary Figure 1 showed the age distributions from the ADNI, AIBL, NACC, and PPMI databases in this study. Participants’ clinical characteristics in the training, internal test, AIBL, NACC, and PPMI test sets were summarized in Tables 1, 2. No significant differences in gender, education, and Mini-Mental State Examination or Montreal Cognitive Assessment between the middle-aged and old-aged groups in all datasets (*p* > 0.05). A significant difference in education was observed between the two groups in the PPMI test set.

**Table 1:** Clinical characteristics in the training and internal test sets from ADNI.

Characteristics	Training set (*n* = 252)		Internal test set (*n* = 108)	
	MAG (*n* = 76)	OAG (*n* = 176)	MAG (*n* = 32)	OAG (*n* = 76)
Gender				
Male	15 (19.74)	49 (27.84)	9 (28.13)	28 (36.84)
Female	61 (80.26)	127 (72.16)	23 (71.87)	48 (63.16)
Age (y)	62.50 (6.58)	70.65 (7.95)	61.35 (6.33)	70.00 (6.77)
Education (y)	16 (4)	16 (3)	16 (4)	16 (3)
MMSE	30 (1)	29 (1.75)	29.5 (1)	29 (1)

Data are expressed as number (percentage) or median (interquartile).

ADNI, Alzheimer’s Disease Neuroimaging Initiative; MAG, middle-aged group; OAG, old-aged group; MMSE, Mini-Mental State Examination.

**Table 2:** Clinical characteristics in the AIBL, NACC, and PPMI test sets.

Characteristics	AIBL test set (*n* = 263)		NACC test set (*n* = 239)		PPMI test set (*n* = 105)	
	MAG (*n* = 51)	OAG (*n* = 212)	MAG (*n* = 77)	OAG (*n* = 162)	MAG (*n* = 66)	OAG (*n* = 39)
Gender						
Male	22 (43.14)	96 (45.28)	30 (38.96)	69 (42.59)	37 (56.06)	27 (69.23)
Female	29 (56.86)	116 (54.72)	47 (61.04)	93 (57.41)	29 (43.94)	12 (30.77)
Age (y)	63.00 (3.00)	73.00 (7.00)	62.00 (8.00)	73.00 (9.00)	59.50 (7.62)	72.00 (6.50)
Education (y)	–	–	16 (5)	16 (4)	16 (4)	18 (3)
MMSE/MoCA	29 (2)^a^	29 (2)^a^	30 (1)^a^	29 (1)^a^	28 (2.25)^b^	28 (2)^b^

Data are expressed as number (percentage) or median (interquartile).

MAG, middle-aged group; OAG, old-aged group; AIBL, Australian Imaging, Biomarker and Lifestyle; NACC, National Alzheimer’s Coordinating Center; PPMI, Parkinson’s Progression Markers Initiative; MMSE, Mini-Mental State Examination; MoCA, Montreal Cognitive Assessment.

^a^Data are MMSE scores from the Alzheimer’s Disease Neuroimaging Initiative database; ^b^data are MoCA scores from the Parkinson’s Progression Markers Initiative database.

Education is not available for the subjects of AIBL database.

### 3.2 VBM and SBM measurement

**Figure 1:**
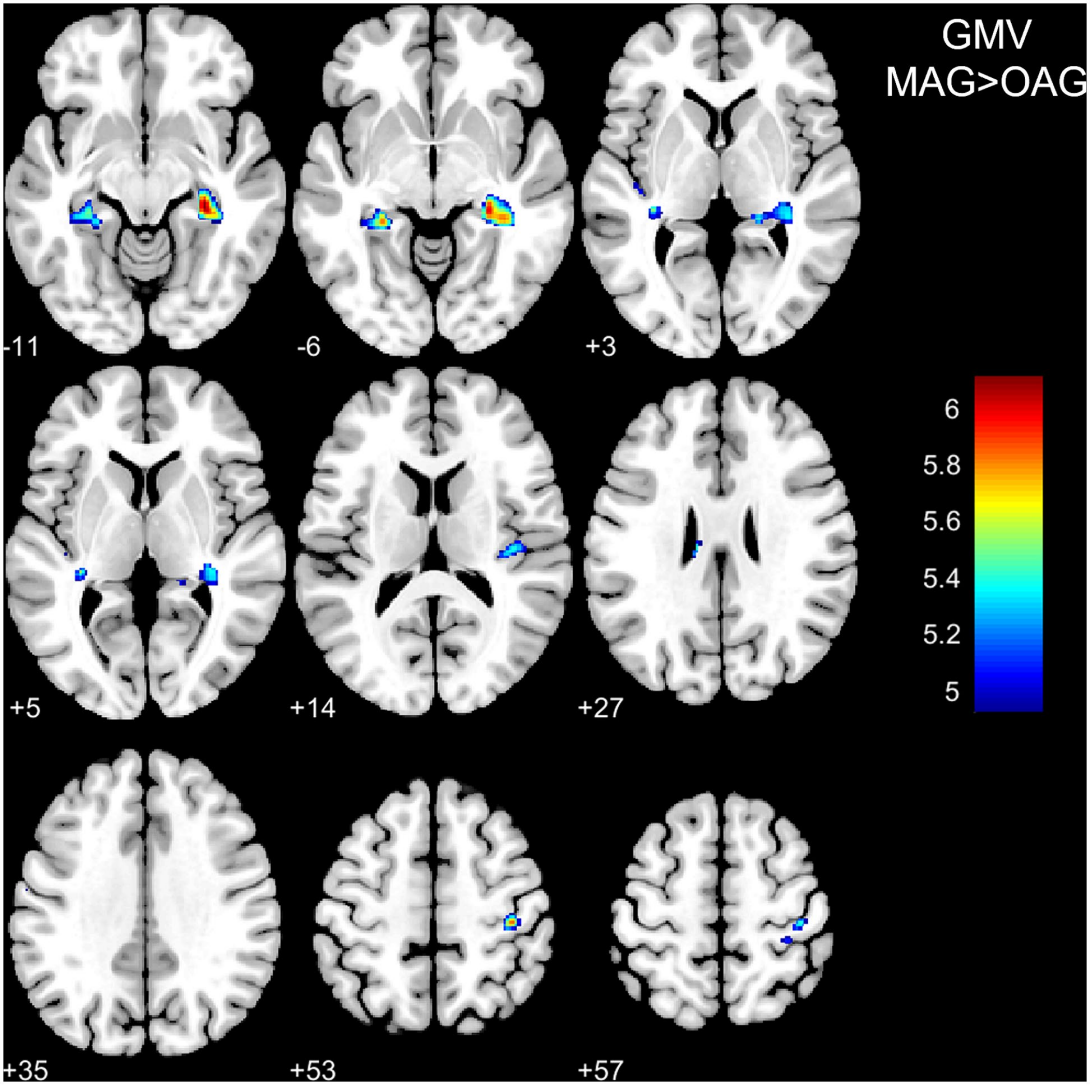
Differences in grey matter volume between the middle-aged and old-aged groups. GMV, grey matter volume; MAG, middle-aged group; OAG, old-aged group. *P* < 0.05, family-wise error-corrected. The color bar represents T values.

**Figure 2:**
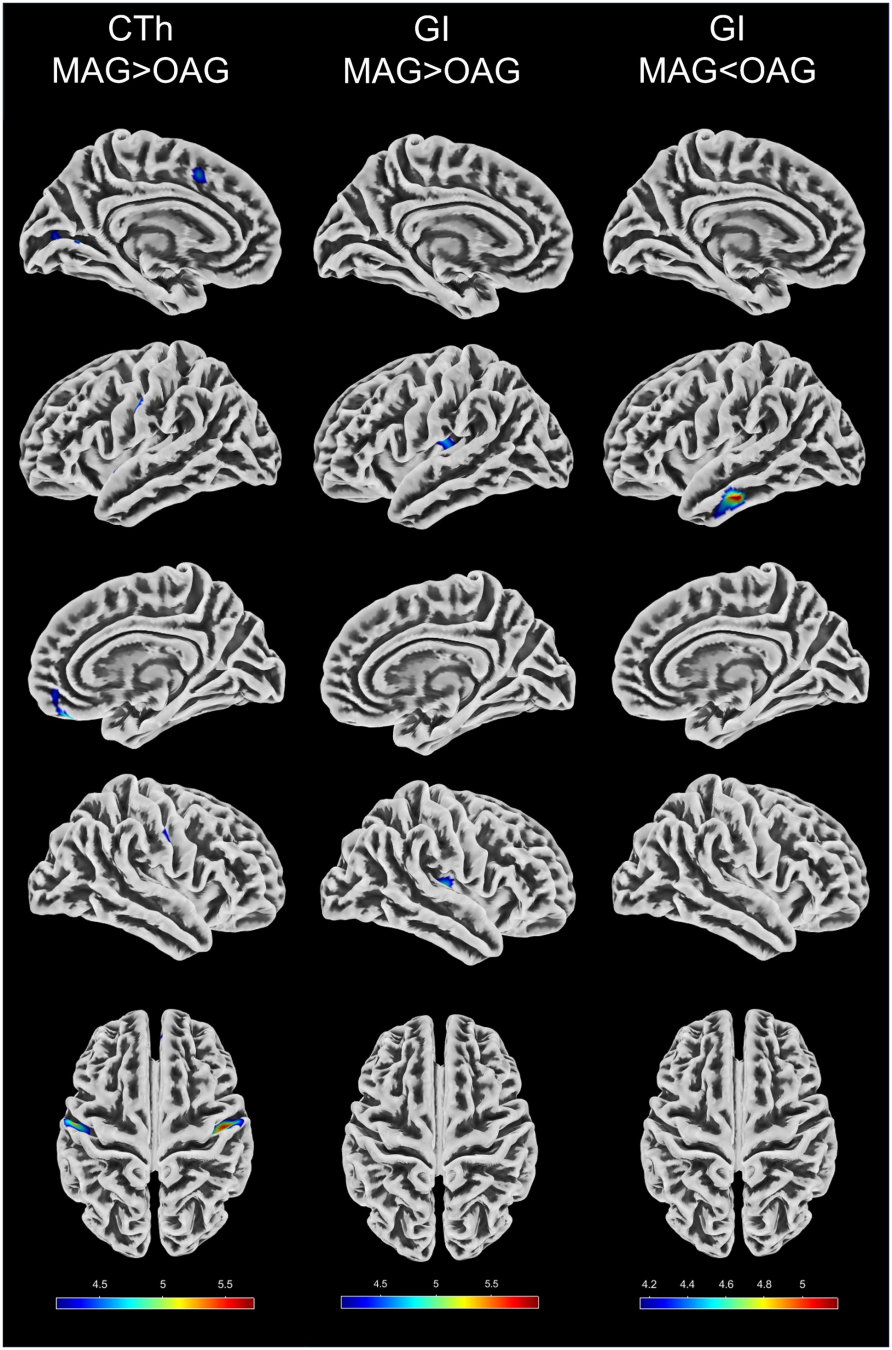
Differences in four parameters of surface-based morphometry between the middle-aged and old-aged groups. CTh, cortical thickness; GI, gyrification index; MAG, middle-aged group; OAG, old-aged group. CTh and GI with the threshold of *p* < 0.05 family-wise error correction. The color bars represent T values.

VBM and SBM results between the middle-aged and old-aged groups were shown in Table 3 and illustrated in Figures 1, 2. In the VBM analysis, the GMV of the old-aged group was significantly lower than that of the middle-aged group within 5 clusters (*p* < 0.05, FWE-corrected). The largest cluster was localized in the right hippocampus (1109 mm^3^; *x* = 30, *y* = −30, *z* = −11; *T* = 6.11; *p* < 0.001). In the SBM analysis, the CTh of the old-aged group was significantly lower than that of the middle-aged group within 8 clusters, which were mostly localized in the bilateral parietal, bilateral frontal, left temporal, and left occipital lobes (*p* < 0.05, FWE-corrected). The GI of the old-aged group was significantly lower than that of the middle-aged group within 2 clusters, of which most were localized in the bilateral insula and temporal lobes, whereas higher GI in the old-aged group was observed within the left middle and inferior temporal regions (*p* < 0.05, FWE-corrected). At the threshold of *p* < 0.05 FWE correction, there was no significant differential cluster in SD and FD between the two groups.

**Table 3:** VBM and SBM results between the middle-aged and old-aged groups.

Cluster size (mm^3^)	MNI coordinates			Side	Brain region	T value	*p*-value
	X	Y	Z				
GMV (MAG > OAG)							
568	−26	−38	−6	L	ParaHippocampal	5.81	0.001
20	−42	−21	3	L	Temporal_Sup	5.05	0.030
1,109	30	−30	−11	R	Hippocampus	6.11	<0.001
124	41	−20	14	R	Heschl	5.38	0.007
449	41	−26	53	R	Postcentral	5.83	0.001
CTh (MAG > OAG)							
183	−47	−17	−3	L	74% Superior temporal	5.73	<0.001
					26% Transverse temporal		
142	−15	−65	2	L	64% Lingual	5.16	0.001
					36% Pericalcarine		
117	−55	−11	30	L	100% Postcentral	5.12	0.001
48	−9	13	47	L	100% Superior frontal	4.62	0.008
217	48	−14	32	R	95% Postcentral	5.48	<0.001
					5% Precentral		
51	5	41	−25	R	100% Medial orbitofrontal	4.88	0.003
18	45	−20	1	R	72% Transverse temporal	4.40	0.020
					28% Superior temporal		
15	16	−63	3	R	100% Lingual	4.32	0.027
GI (MAG > OAG)							
253	−36	−27	4	L	72% Insula	4.96	0.002
					11% Transverse temporal		
					11% Postcentral		
					4% Precentral		
					2% Superior temporal		
181	38	−24	1	R	69% Insula	5.92	<0.001
					21% Superior temporal		
					10% Transverse temporal		
GI (MAG < OAG)							
129	−56	−19	−25	L	59% Middle temporal	5.19	0.001
					41% Inferior temporal		

MAG, middle-aged group; OAG, old-aged group; MNI, Montreal Neurological Institute; GMV, grey matter volume; CTh, cortical thickness; GI, gyrification index; L, left; R, right.

The brain regions of GMV were from Anatomical Automatic Labeling (AAL) Atlas, and the brain regions of CTh and GI were from Desikan-Killiany (DK40) Atlas.

*P* < 0.05, family-wise error-corrected.

### 3.3 Model performance comparison

Table 4 listed 13 differential brain regions with potential for building the VBM-SBM model in the training set. After the univariate and multivariable logistic regression analysis, the model was finally developed with the GMV of right hippocampus (OR = 0.903; 95% CI: 0.825, 0.988; *p* = 0.026), the CTh of left lingual (OR = 0.649; 95% CI: 0.495, 0.850; *p* = 0.002), and the GI of left insula (OR = 0.970; 95% CI: 0.948, 0.992; *p* = 0.008).

**Table 4:** Logistic regression analysis of differential brain regions associated with aging.

Per 0.1 increase	Side	Brain region	Univariable		Multivariable	
			OR (95% CI)	*P*-value	OR (95% CI)	*P*-value
GMV	L	ParaHippocampal	0.987 (0.914, 1.066)	0.744	NA	NA
	R	Hippocampus	0.861 (0.793, 0.935)	<0.001	0.903 (0.825, 0.988)	0.026
	R	Heschl	0.643 (0.494, 0.835)	0.001	NA	NA
	R	Postcentral	0.977 (0.952, 1.004)	0.095	NA	NA
CTh	L	Superior temporal	0.655 (0.519, 0.826)	<0.001	NA	NA
	L	Lingual	0.651 (0.505, 0.840)	0.001	0.649 (0.495, 0.850)	0.002
	L	Postcentral	0.716 (0.570, 0.899)	0.004	NA	NA
	R	Postcentral	0.709 (0.568, 0.883)	0.002	NA	NA
	R	Medial orbitofrontal	0.665 (0.494, 0.896)	0.007	NA	NA
GI	L	Insula	0.967 (0.947, 0.987)	0.001	0.970 (0.948, 0.992)	0.008
	R	Insula	0.972 (0.954, 0.991)	0.005	NA	NA
	L	Middle temporal	1.022 (0.998, 1.048)	0.076	NA	NA
	L	Inferior temporal	1.020 (0.993, 1.048)	0.140	NA	NA

OR, odds ratio; CI, Confidence interval; NA, not applicable; GMV, grey matter volume; CTh, cortical thickness; GI, gyrification index; L, left; R, right.

The brain regions of GMV were from Anatomical Automatic Labeling (AAL) Atlas, and the brain regions of CTh and GI were from Desikan-Killiany (DK40) Atlas.

*P* < 0.05 indicates statistical significance.

**Figure 3:**
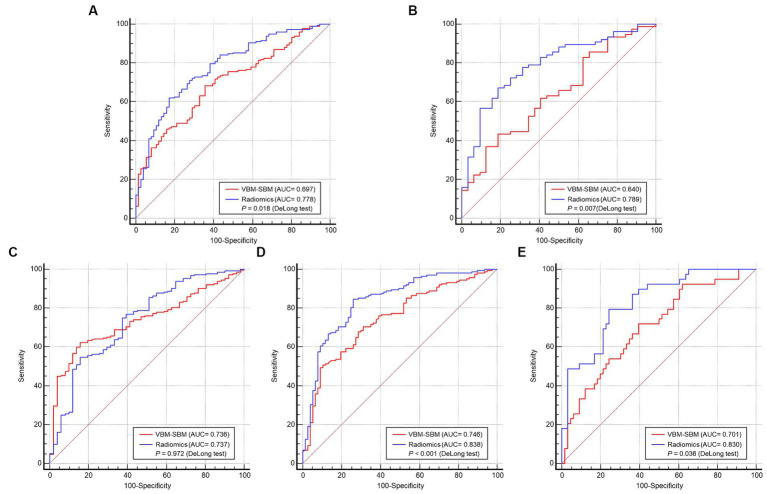
Performance comparisons between the training set **(A)**, internal test set **(B)**, AIBL test set **(C)**, NACC test set **(D)**, PPMI test set **(E)**. VBM, Voxel-based morphometry; SBM, surface-based morphometry; AUC, area under the receiver operating characteristic curve.

1,132 radiomics features were extracted from the GM of each participant. Supplementary Figure 2 showed the data distributions from the four databases before and after the ComBat method. Through features removement, selection, classification, and 5-fold cross-validation, four radiomics features were ultimately retained for model construction. Supplementary Figure 3 showed the four-feature model according to one-standard error rule in the cross-validation set. The features were log-sigma-2-0-mm-3D_firstorder_Mean, log-sigma-2-0-mm-3D_firstorder_Median, log-sigma-3-0-mm-3D_glszm_ZoneEntropy, wavelet-HHL_firstorder_Median. The definitions of the four features were summarized in the Supplementary Method 1. The AUCs of the VBM-SBM model and radiomics model were 0.697 and 0.778 in the training set (*p* = 0.018), 0.640 and 0.789 in the internal test set (*p* = 0.007), 0.736 and 0.737 in the AIBL test set (*p* = 0.972), 0.746 and 0.838 in the NACC test set (*p* < 0.001), and 0.701 and 0.830 in the PPMI test set (*p* = 0.036). The detailed comparisons of model performance were shown in Figure 3 and Tables 5, 6.

**Table 5:** Performance comparisons of models in the training and internal test sets from ADNI.

	Training set (*n* = 252)		Internal test set (*n* = 108)	
	Model 1	Model 2	Model 1	Model 2
AUC (95% CI)	0.697 (0.636, 0.753)	0.778 (0.717, 0.839)	0.640 (0.542, 0.730)	0.789 (0.694, 0.877)
Sensitivity	0.682	0.619	0.434	0.671
Specificity	0.645	0.829	0.813	0.813
Accuracy	0.671	0.683	0.546	0.713
PPV	0.816	0.893	0.846	0.895
NPV	0.467	0.485	0.377	0.510

Model 1, VBM-SBM model; Model 2, radiomics model; AUC, area under the receiver operating characteristic curve; CI, Confidence interval; PPV, positive predictive value; NPV, negative predictive value.

**Table 6:** Performance comparisons of models in the AIBL, NACC, and PPMI test sets.

	AIBL test set (*n* = 263)		NACC test set (*n* = 239)		PPMI test set (*n* = 105)	
	Model 1	Model 2	Model 1	Model 2	Model 1	Model 2
AUC (95% CI)	0.736 (0.678, 0.788)	0.737 (0.652, 0.816)	0.746 (0.686, 0.800)	0.838 (0.778, 0.889)	0.701 (0.604, 0.787)	0.830 (0.751, 0.905)
Sensitivity	0.623	0.547	0.494	0.846	0.718	0.795
Specificity	0.843	0.843	0.909	0.740	0.606	0.758
Accuracy	0.665	0.605	0.628	0.812	0.648	0.771
PPV	0.943	0.936	0.920	0.873	0.519	0.660
NPV	0.350	0.309	0.461	0.695	0.784	0.862

Model 1, VBM-SBM model; Model 2, radiomics model; AUC, area under the receiver operating characteristic curve; CI, Confidence interval; PPV, positive predictive value; NPV, negative predictive value.

### 3.4 Associations between age, VBM-SBM parameters, and radiomics features

**Figure 4:**
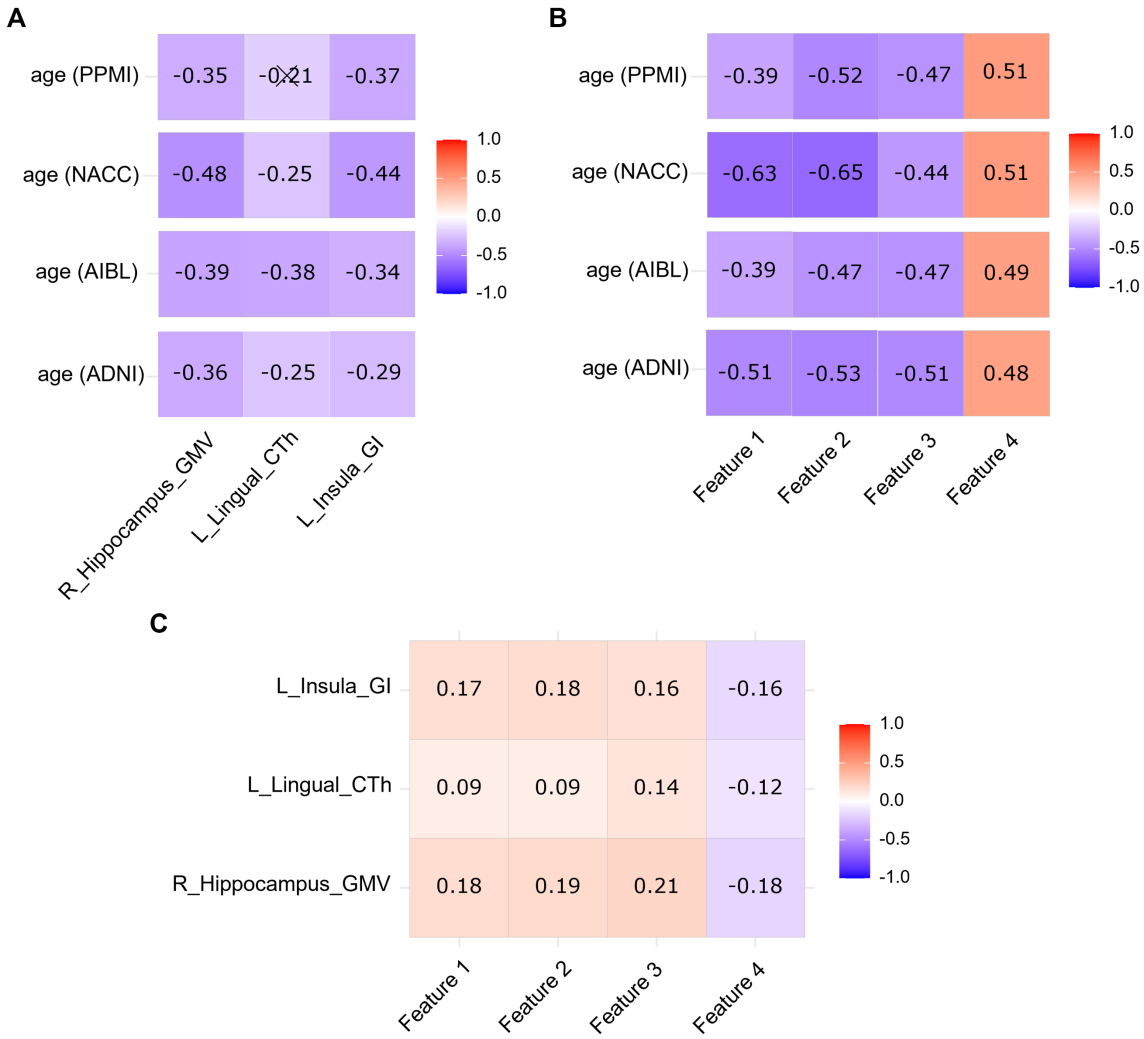
Correlation heatmap between age and VBM-SBM parameters **(A)**, correlation heatmap between age and radiomics features **(B)**, and correlation heatmap between VBM-SBM parameters and radiomics features **(C)** using Spearman correlation coefficients. L, left; R, right; GMV, grey matter volume; CTh, cortical thickness; GI, gyrification index. Feature 1, log-sigma-2-0-mm-3D_firstorder_Mean; Feature 2, log-sigma-2-0-mm-3D_firstorder_Median; Feature 3, log-sigma-3-0-mm-3D_glszm_ZoneEntropy; Feature 4, wavelet-HHL_firstorder_Median. Cross markers indicate no significant correlations (*p* > 0.05). The color bar shows the positive and negative of the correlation coefficient as well as the magnitude.

Figure 4A showed the correlations between age and VBM-SBM parameters in the ADNI, AIBL, NACC, and PPMI datasets. Age was negatively correlated with the GMV of right hippocampus, the CTh of left lingual, and the GI of left insula in all datasets (*p* < 0.05), except for the CTh of left lingual in the PPMI database (*p* > 0.05). Figure 4B showed the correlations between age and radiomics features in the ADNI, AIBL, NACC, and PPMI datasets. Age was negatively correlated with log-sigma-2-0-mm-3D_firstorder_Mean, log-sigma-2-0-mm-3D_firstorder_Median, log-sigma-3-0-mm-3D_glszm_ZoneEntropy, and positively correlated with wavelet-HHL_firstorder_Median in all datasets (*p* < 0.05). VBM-SBM parameters had significant correlations with radiomics features (*p* < 0.05; Figure 4C).

## 4 Discussion

VBM-SBM and radiomics methods are commonly used to analyze brain imaging from various perspectives. However, there has been no research comparing the two methods to explore their performance, associations, strengths, and applicable situations. In the current study, we built two normal brain aging models based on the GM of CN individuals, including a VBM-SBM model and a radiomics model. The radiomics model had significantly higher AUCs than the VBM-SBM model in the training, internal test, NACC test, and PPMI test sets. The correlations between age and four radiomics features were generally stronger than correlations between age and three VBM-SBM parameters, and weak relationships were found between the VBM-SBM parameters and radiomics features.

Previous researchers have used VBM and SBM to explore their relationships with age, however, this study applied the differential brain regions to the construction of a brain aging model. It was reported that the results of morphometric analysis varied with populations and thresholds, but there were still some frequently mentioned (Goto et al., 2022). In the literature on VBM research, it was found that the GMV of multiple brain regions significantly decreases with age, such as the temporal, occipital, and parietal lobes (Fleischman et al., 2014; Zheng et al., 2019). In our study, 5 regions from the temporal and parietal had significant differences between the middle-aged and old-aged groups. Finally, the right hippocampus, the largest cluster size, was selected to construct the model. In the SBM analysis, research on CTh showed that cortical thinning was more widespread than GMV loss (Fleischman et al., 2014). We found that the CTh of 12 regions had significant decreases with age, which could be observed in the temporal, occipital, parietal, and frontal lobes. This finding was consistent with previous research (Fjell et al., 2009). After the selection through logistic regression, the left lingual participated in the model construction. GI, a quantification of cortex folding structure, changes with aging and relates to the development of cognitive function across the lifespan (Cao et al., 2017). Studies have reported that global cortical gyrification gradually decreases with age, while significantly differential regions for local cortical gyrification vary in sample size and age distribution (Lamballais et al., 2020; Madan, 2021). We found the 8 regions of the old-aged group had a significantly lower GI than that of the middle-aged group, and the bilateral insular regions accounted for a large proportion. The left insula was finally included to the model. Higher GIs in 2 regions from the temporal lobe of the old-aged group were also observed in this study.

The ranges of AUCs of the VBM-SBM model and radiomics model were 0.640 ~ 0.746 and 0.737 ~ 0.838, respectively. In the training set, the radiomics model had an AUC of 0.778, which was significantly higher than the VBM-SBM model (AUC = 0.697). For the test sets, the VBM-SBM model had an AUC range of 0.701 ~ 0.746 in the three external test sets, but an AUC of 0.640 in the internal test set. The radiomics model had an AUC range of 0.737 ~ 0.838 in four test sets. These indicate that the radiomics model can better distinguish the age-related groups and have higher generalization; however, it suffers from a deficiency in biological interpretability, and the selected features have some challenges in clinical application. For the VBM-SBM model, it can locate important brain regions and the parameters are often used in clinical practice, but its performance is slightly weak, and the processing is relatively cumbersome and time-consuming. Overall, both approaches have their advantages and disadvantages, and investigators can choose based on their study objectives. For researchers prioritizing performance and generalization, radiomics offers a good choice, while those emphasizing interpretability and clinical practice may find VBM-SBM more suitable.

By observing the correlations between age and VBM-SBM parameters, and age and radiomics features, we noted that the correlations between age and the four radiomics features were generally stronger than the correlations between age and the three VBM-SBM parameters. This suggests that the radiomics features have closer relationships with age than the VBM-SBM parameters in brain aging analysis. We also compared the relationships between the VBM-SBM parameters and radiomics features, the results showed that though the indicators from the two methods had significant correlations, the associations were relatively weak, which means that VBM-SBM and radiomics analyze brain aging from different dimensions and perform their respective functions.

Several limitations in our investigation need to be acknowledged. Firstly, the retrospective data were from four databases, which had inherent biases in selecting subjects and might not represent real-world situations. Secondly, the age span between groups was relatively narrow, which could limit the transferability of the model performance. Thirdly, the selection thresholds of VBM-SBM parameters and radiomics features were based on the current study, which can be varied according to different research goals and situations. Finally, there are many methods and software available for morphometry and radiomics analysis of the brain. Therefore, the results of the model performance might change under different experimental conditions.

## 5 Conclusion

In conclusion, the radiomics model performed better than the VBM-SBM model. Radiomics focuses on the generalization and VBM-SBM has the interpretability. VBM-SBM and radiomics analyze brain aging from different dimensions and perform their respective functions.

## Data availability statement

Publicly available datasets were analyzed in this study. This data can be found at: the Alzheimer’s Disease Neuroimaging Initiative (ADNI) database (https://adni.loni.usc.edu/), the Australian Imaging, Biomarker and Lifestyle (AIBL) database (https://aibl.org.au/), the National Alzheimer’s Coordinating Center (NACC) database (https://naccdata.org/), and the Parkinson’s Progression Markers Initiative (PPMI) database (https://www.ppmi-info.org/).

## Ethics statement

The studies involving humans were approved by the Ethics Committees of Alzheimer’s Disease Neuroimaging Initiative (ADNI), Australian Imaging, Biomarker and Lifestyle (AIBL), National Alzheimer’s Coordinating Center (NACC), and Parkinson’s Progression Markers Initiative (PPMI). The studies were conducted in accordance with the local legislation and institutional requirements. The participants provided their written informed consent to participate in this study.

## Author contributions

YY: Conceptualization, Data curation, Formal analysis, Methodology, Writing – original draft, Writing – review & editing. XH: Conceptualization, Data curation, Methodology, Writing – review & editing. YX: Data curation, Formal analysis, Writing – review & editing. JP: Formal analysis, Visualization, Writing – review & editing. FZ: Formal analysis, Writing – review & editing. YS: Conceptualization, Data curation, Formal analysis, Funding acquisition, Methodology, Supervision, Writing – review & editing.

## Footnotes

^1^ https://adni.loni.usc.edu/

^2^ https://aibl.org.au/

^3^ https://naccdata.org/

^4^ https://www.ppmi-info.org/

^5^ https://www.fil.ion.ucl.ac.uk/spm/software/spm12/

^6^ https://neuro-jena.github.io/cat//

^7^ https://people.cas.sc.edu/rorden/mricron/dcm2nii.html

^8^ https://www.alivelearn.net/xjview/download-link/

^9^ https://dbm.neuro.uni-jena.de/cat12/CAT12-Manual-old.pdf

^10^ http://www.itksnap.org
