# Supplementary material for: Comparison between morphometry and radiomics: detecting normal brain aging based on grey matter
Source: Front Aging Neurosci. 2024 Apr 15;16:1366780. doi: 10.3389/fnagi.2024.1366780 (PMC11056505; doi:10.3389/fnagi.2024.1366780)
Supplement: Supplementary file 1 [file Data_Sheet_1.docx]

**Supplementary Method** **1** Definitions of the four features

The four radiomics features included in the model are as follows:

log-sigma-2-0-mm-3D_firstorder_Mean

log-sigma-2-0-mm-3D_firstorder_Median

log-sigma-3-0-mm-3D_glszm_ZoneEntropy

wavelet-HHL_firstorder_Median

The naming method of the 4 features was: **Image types_Feature classes_Specific features**

1. **Image types**

Many filters are provided in the Pyradiomics. These are the image types (either the original image or derived images using filters) that can be used to extract features. The image types concerned in this study are:

-**LoG**: Laplacian of Gaussian filter emphasizes areas of gray level change, where sigma defines how coarse the emphasized texture should be. A low sigma emphasizes fine textures (change over a short distance), whereas a high sigma emphasizes coarse textures (gray level change over a large distance).

-**Wavelet**: Wavelet filtering, yields 8 decompositions per level (all possible combinations of applying either a High or a Low pass filter in each of the three dimensions, including LLH, LHL, LHH, HLL, HLH, HHL, HHH, LLL).

1. **Feature classes**

-**First-order**: First-order features describe the distribution of voxel intensities within the image region defined by the mask through commonly used and basic metrics.

-**GLSZM**: One of the texture features. A Gray Level Size Zone Matrix (GLSZM) quantifies gray level zones in an image. A gray level zone is defined as the number of connected voxels that share the same gray level intensity.

1. **Specific features**

-**Mean**: The average gray level intensity within the ROI.

**-Median**: The median gray level intensity within the ROI.

-**Zone Entropy**: It measures the uncertainty/randomness in the distribution of zone sizes and gray levels. A higher value indicates more heterogeneity in the texture patterns.

**
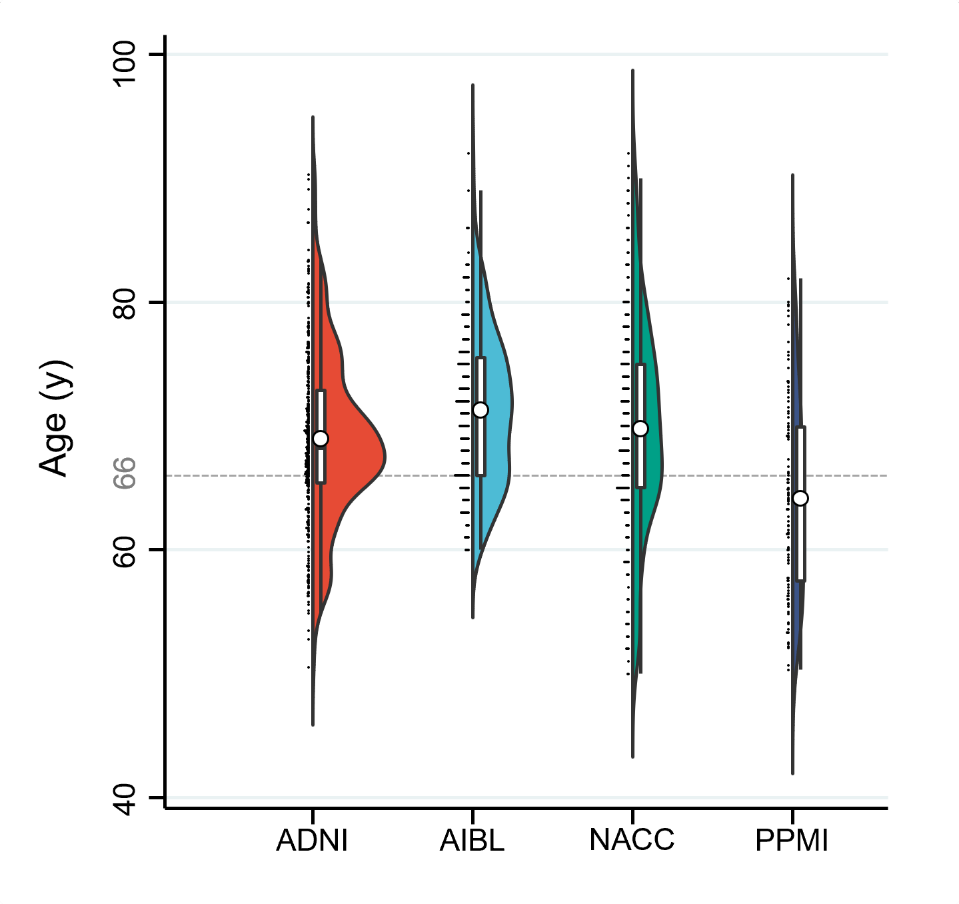
**

**Supplementary Figure 1** The age distributions from the four databases in this study. ADNI, Alzheimer’s Disease Neuroimaging Initiative; AIBL, Australian Imaging, Biomarker and Lifestyle; NACC, National Alzheimer’s Coordinating Center; PPMI, Parkinson’s Progression Markers Initiative. Ages are integers in the AIBL and NACC databases, and ages are accurate to one decimal place in the ADNI and PPMI databases.

**
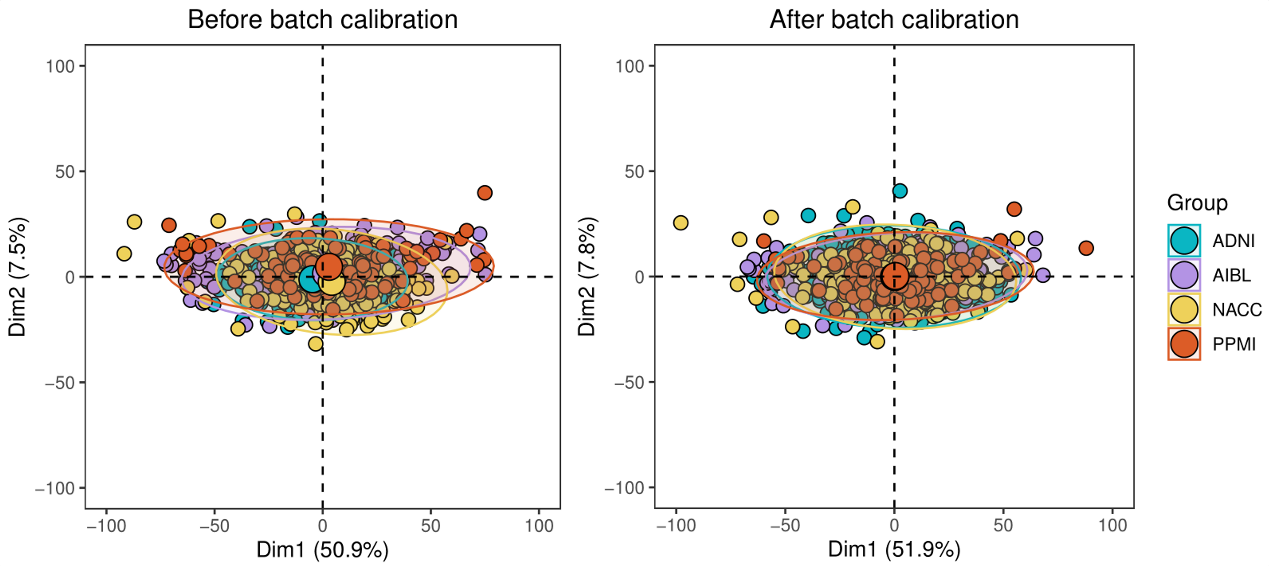
**

**Supplementary Figure 2** The data distributions from the four databases before and after the ComBat method. ADNI, Alzheimer’s Disease Neuroimaging Initiative; AIBL, Australian Imaging, Biomarker and Lifestyle; NACC, National Alzheimer’s Coordinating Center; PPMI, Parkinson’s Progression Markers Initiative.

**
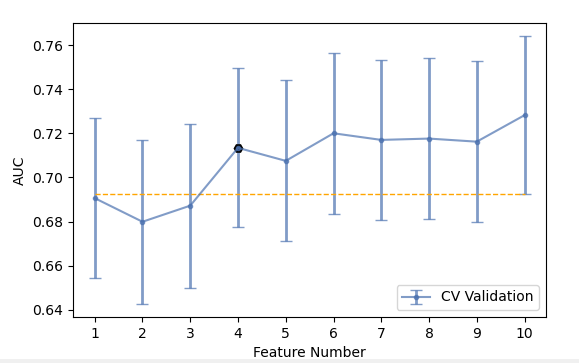
**

**Supplementary Figure 3** The four-feature model according to the one-standard error rule in the cross-validation set. AUC, area under the receiver operating characteristic curve.
